# Daytime soybean transcriptome fluctuations during water deficit stress

**DOI:** 10.1186/s12864-015-1731-x

**Published:** 2015-07-07

**Authors:** Fabiana Aparecida Rodrigues, Renata Fuganti-Pagliarini, Juliana Marcolino-Gomes, Thiago Jonas Nakayama, Hugo Bruno Correa Molinari, Francisco Pereira Lobo, Frank G Harmon, Alexandre Lima Nepomuceno

**Affiliations:** Brazilian Agricultural Research Corporation- Embrapa Soybean, Embrapa Soybean- Rod. Carlos João Strass, s/n, Londrina, 86001-970 PR Brazil; Department of Biology, State University of Londrina, Londrina, PR Brazil; Department of Crop Science, Federal University of Viçosa, Viçosa, MG Brazil; Genetics and Biotechnology Laboratory, Embrapa Agroenergy (CNPAE), Brasília, DF Brazil; Brazilian Agricultural Research Corporation-Embrapa Agricultural Informatics, Campinas, SP Brazil; Plant Gene Expression Center, USDA-ARS, Albany, CA USA; Department of Plant and Microbial Biology, UC Berkeley, Berkeley, CA USA; Embrapa LABEX US Plant Biotechnology at ARS/USDA Plant Gene Expression Center, Albany, CA USA

**Keywords:** Abiotic stress, Daily oscillation, *diel* regulation, Drought, *Glycine max*, Plant metabolism

## Abstract

**Background:**

Since drought can seriously affect plant growth and development and little is known about how the oscillations of gene expression during the drought stress-acclimation response in soybean is affected, we applied Illumina technology to sequence 36 cDNA libraries synthesized from control and drought-stressed soybean plants to verify the dynamic changes in gene expression during a 24-h time course. Cycling variables were measured from the expression data to determine the putative circadian rhythm regulation of gene expression.

**Results:**

We identified 4866 genes differentially expressed in soybean plants in response to water deficit. Of these genes, 3715 were differentially expressed during the light period, from which approximately 9.55 % were observed in both light and darkness. We found 887 genes that were either up- or down-regulated in different periods of the day. Of 54,175 predicted soybean genes, 35.52 % exhibited expression oscillations in a 24 h period. This number increased to 39.23 % when plants were submitted to water deficit. Major differences in gene expression were observed in the control plants from late day (ZT16) until predawn (ZT20) periods, indicating that gene expression oscillates during the course of 24 h in normal development. Under water deficit, dissimilarity increased in all time-periods, indicating that the applied stress influenced gene expression. Such differences in plants under stress were primarily observed in ZT0 (early morning) to ZT8 (late day) and also from ZT4 to ZT12. Stress-related pathways were triggered in response to water deficit primarily during midday, when more genes were up-regulated compared to early morning. Additionally, genes known to be involved in secondary metabolism and hormone signaling were also expressed in the dark period.

**Conclusions:**

Gene expression networks can be dynamically shaped to acclimate plant metabolism under environmental stressful conditions. We have identified putative cycling genes that are expressed in soybean leaves under normal developmental conditions and genes whose expression oscillates under conditions of water deficit. These results suggest that time of day, as well as light and temperature oscillations that occur considerably affect the regulation of water deficit stress response in soybean plants.

**Electronic supplementary material:**

The online version of this article (doi:10.1186/s12864-015-1731-x) contains supplementary material, which is available to authorized users.

## Background

Soybean is one of the most important crops in the world. Overall yield is highly affected by water deficit stress, particularly when the stress occurs during flowering and early pod expansion [1]. To overcome the water limitation and facilitate the continued expansion of soybean productivity and crop improvement, implementation of modern biotechnology such as genetic engineering of plants to produce drought-tolerant cultivars, rises as a potential solution [2]. However, the achievement of such a goal is highly dependent on the elucidation of the molecular mechanisms of drought tolerance and their interaction with environmental cues. A better understanding of these aspects would help identify candidate genes for genetic engineering of improved stress-tolerant crops [3].

To better fit to the surrounding environmental conditions, such as season and light/darkness and temperature variations, it is known that plants, as sessile organisms, coordinate and regulate their metabolism and physiology through an endogenous circadian clock. This clock drives rhythms at the molecular and cellular levels and, thus, temporally regulates plant physiology and behavior to anticipate changes in the environment [4]. According to Khan et al. [5], the consequence of proper clock and environment synchronization is optimized fitness. However, abiotic stresses, such as drought, change the clock synchrony, altering the circadian rhythm in response to dehydration [6].

Genome-wide analysis of mRNA expression shows daily oscillation coordinated by circadian rhythms, which, in turn, regulate various biological processes [7–9], such as seed dormancy and germination [10], hormone metabolism [11–13], and grapevine fruit ripening [14], among other processes [10]. Gene expression regulated by the circadian rhythm also has been observed in some plant responses to abiotic stress [15–17]. In the process of cold acclimation in *Arabidopsis*, gene expression was affected by time of day, revealing an interaction between cold and diurnal regulation that drives transcriptome changes [15]. Furthermore, time of day also was an important cue for triggering changes in the *Populus* transcriptome [16] and in *Arabidopsis* plants in response to soil drying [17]. In soybean, evidence also suggests that the circadian rhythm plays a role in regulating genes involved in developing seeds [18].

Although soybean is one of the most studied crops using molecular biology tools, little is known about how daily oscillations of gene expression are affected by drought stress during the survival or acclimation response. The regulation of a proline-rich-protein gene, induced under drought and salt stresses in specific tissues of soybean seedlings, was demonstrated to be circadian-controlled [19]. Considering the dynamic changes of plant metabolism that occur to coordinate the daily variation in light and temperature, the evaluation of gene expression at different time periods of the day becomes valuable for identifying times during which key genes might be most influential in the defense response [17]. In *Arabidospis*, hormone-related genes, specifically the abscisic acid (ABA)-responsive genes, are correlated with diurnal oscillations [12]. The functional roles of some representatives, like the dehydrins class and the *rd29A*, or the cold-regulated *COR15B/15A* and the low temperature-induced *LTI30*, may be related to responses to water deficit and low temperature, respectively [12].

Recently, our research team has demonstrated how drought impacts diurnal oscillation of both drought-responsive and circadian clock genes in soybean [20]. Drought stress induced marked reduction in gene expression levels of several circadian clock-like components, such as *GmLCL1*-, *GmELF4*-, and *GmPRR*-like genes. The same conditions produced a phase advance of expression for the *GmTOC1-*, *GmLUX*- and *GmPRR7*-like genes. Similarly, the daily oscillation pattern of the soybean drought-responsive genes *DREB-*, *bZIP-*, *GOLS-*, *RAB18-* and *Remorin-*like changed significantly following plant exposure to water deficit.

With the goal of detecting genome-wide transcriptome changes during the entire period that plants were exposed to moderate water deficit and if such variations occurred in a time-of-day-dependent manner, we analyzed multiple time points in a *diel* period. Here, we present a survey of soybean genes expressed under stress and their daily oscillation waveforms during a 24-h time course. We also determined their abundance, and we suggest the putative biological roles of these differentially expressed genes.

## Results

### Genes differentially expressed in response to water deficit

The expression pattern of soybean genotype BR16, previously characterized as drought sensitive [21], was evaluated under normal and water deficit conditions, during a 24-h time course. To identify differentially expressed genes (DEGs) in response to water deficit treatment, we applied a stringent statistical test to determine whether genes were either up- or down-regulated compared to those of plants under optimal hydration conditions. The resulting ratio represented the fold-change (fc) for each gene. To avoid false positives and reliably identify the most significant changes in gene expression, only genes with fc ≤ -2 (down) and ≥ 2 (up) were considered. We also applied a stringent statistical significance cutoff (adjusted *p*-value ≤ 0.01) to improve confidence (Additional file 1).

**Fig. 1:**
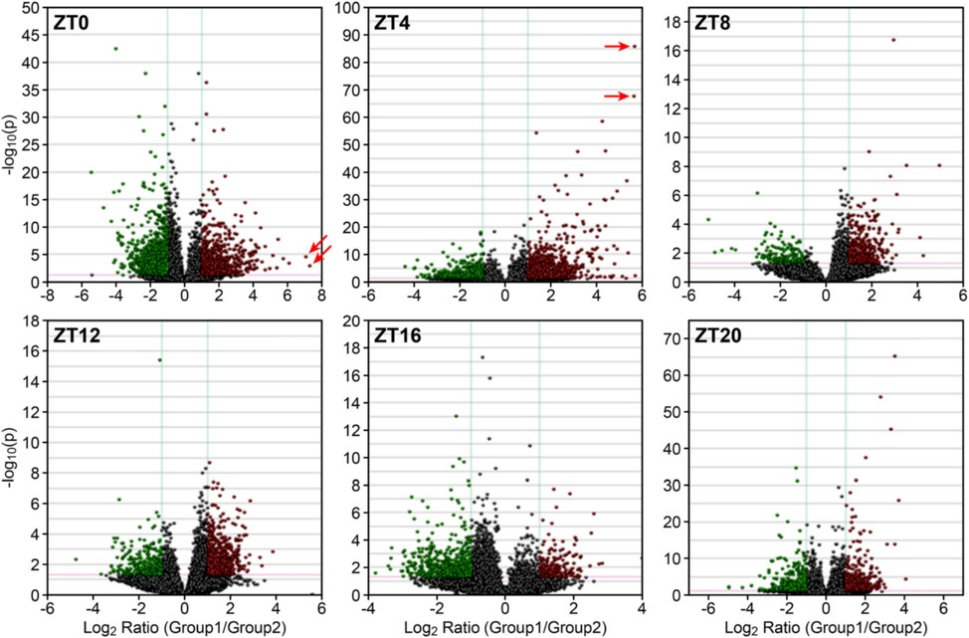
Volcano plots. Expression data were plotted on a log_2_ scale (x-axis) versus a -log_10_ transformation of the *p*-value (y-axis). Datasets were filtered to remove genes with low expression levels (blue lines from -1 to 1 on the x-axis), and a significance cut off (*p* < 0.01) was applied (red line on the y-axis). ZT0 (*n* = 2218; 582 up and 1636 down); ZT4 (*n* = 1290; 856 up and 434 down); ZT8 (*n* = 207; 175 up and 32 down); ZT12 (*n* = 497; 448 up and 49 down); ZT16 (*n* = 175; 73 up and 102 down); and ZT20 (*n* = 479; 273 up and 206 down). Arrows indicate the highest expression in ZT0 (*Glyma18g43980* and *Glyma10g38050*) and the highest confidence in ZT4 (*Glyma03g24320* and *Glyma17g13720*)

There were larger sets of differentially expressed genes in ZT0 (*n* = 2218) and ZT4 (*n* = 1290) compared to the other periods. In ZT0, the majority of genes (73.76 %) were down-regulated under water deficit stress condition, this is in contrast to the other periods (ZT4, ZT8, ZT12, and ZT20), in which genes were primarily up-regulated (66.35 %, 84.54 %, 90.14 %, and 56.99 %, respectively) (Fig. 1, Additional file 1). In general, a moderate expression ratio was detected for most genes, but high fold-changes for some genes were observed in the ZT0 and ZT4 periods. The highest differential expression was detected in ZT0 for *Glyma18g43980* (156.41 fc), which codes for a UDP-glucosyl transferase 73B5, and for *Glyma10g38050* (134.53 fc), a CAP160 protein. In ZT4, the highest confidence levels were found for *Glyma03g24320*, a gene related to the fatty acid hydroxylase superfamily and for *Glyma17g13720*, with unknown function (Fig. 1, Additional file 1).

**Fig. 2:**
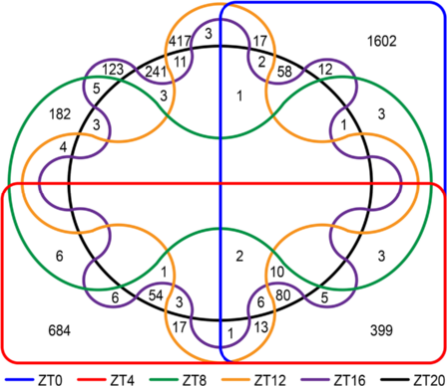
Edwards’s diagram. Number of genes that were differentially expressed in each time period or in more than one period. ZT0 (*n* = 2218); ZT4 (*n* = 1290); ZT8 (*n* = 207); ZT12 (*n* = 497); ZT16 (*n* = 175); and ZT20 (*n* = 479)

In this study, we found 4866 genes that were differentially expressed in soybean plants in response to moderate water deficit during a 24-h time course (Fig. 1, Additional file 1). These genes represented 8.98 % of the 54,175 predicted genes in the Glyma 1.1 soybean genome assembly [22]. However, considering that some genes (*n* = 887; Fig. 2) were either up- or down- regulated during multiple time periods, 3979 genes (approximately 7.34 % of the genome) were uniquely expressed in response to water deficit. The number of genes exclusively expressed in each time period and those observed in multiple ones are presented in Fig. 2 and in Additional file 2. Most of the DEGs were identified in the early and late morning periods (ZT0 and ZT4). Approximately 72.2 % (ZT0; 279 genes up-regulated and 1323 genes down-regulated) and 53 % (ZT4; 502 genes up- and 182 genes down-) were expressed exclusively in one period, whereas others were detected in both periods (ZT0–ZT4; *n* = 399) (Fig. 2, Additional files 1 and 2). Fewer genes were differentially expressed in ZT8 (*n* = 207; 175 genes up- and 32 genes down-) and ZT16 (*n* = 175; 73 genes up- and 102 genes down-) in response to water deficit (Additional file 1). However, most of these genes were exclusively detected in ZT8 (87.9 %; 155 genes up- and 27 genes down-) and ZT16 (70 %; 53 genes up- and 70 genes down-), respectively (Fig. 2, Additional file 2). Similarly, in ZT12 (*n* = 497; 448 genes up- and 49 genes down-) (Additional file 1), 83.9 % of the genes (379 up- and 38 down-) were found only in this time period (Fig. 2, Additional file 2). At pre-dawn (ZT20), 479 genes (273 up- and 206 down-) were identified as differentially expressed (Additional file 1), from which 144 up- (52.7 %) and 97 down-regulated (47 %) genes were observed exclusively in this time period (Fig. 2, Additional file 2).

**Fig. 3:**
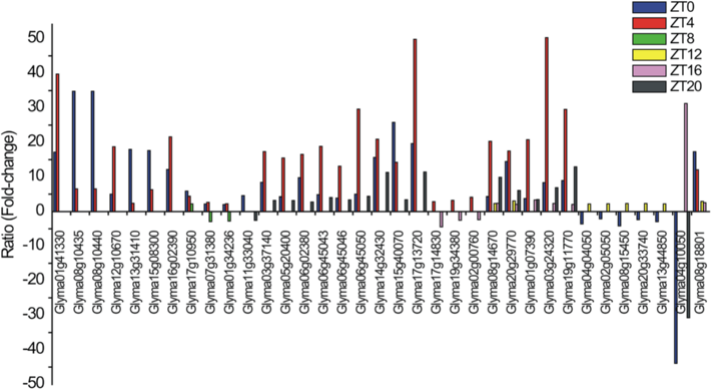
Genes differentially expressed in different time periods with a diverse expression pattern. Gene expression was analyzed with edgeR statistical test to determine a ratio of expression (fold-change) between control and drought-stressed plants. The y-axis represents the fold-change value. All data shown are statistically significant (adjusted *p*-value of *p* ≤ 0.01)

In general, genes differentially expressed in ZT0–ZT4 kept their expression profiles during both time periods, either increasing (*Glyma01g41330* [coding for expansin-like B1], *Glyma12g10670* [Ras-related small GTP-binding family protein], and *Glyma16g02390* [homeobox 7]) or decreasing (*Glyma08g10435*, *Glyma08g10440*, *Glyma13g31410* [aluminium-induced protein with YGL and LRDR motifs], and *Glyma15g08300* [dormancy-associated protein-like 1]) their differential expression levels from early to late morning (Fig. 3). Three up-regulated genes that were detected during morning (ZT0–ZT4) also exhibited decreased differential expression (*Glyma17g10950* [expansin A15]) or changed their profiles (*Glyma07g31380* [cytochrome P450] and *Glyma01g34236*) at the late day (ZT8) (Fig. 3). We found 58 genes identified as differentially expressed in both ZT0 (early morning) and ZT20 (dark period) (Fig. 2). Dynamic changes in expression were not observed for these genes, with the exception of *Glyma11g33040*, which codes for an oxidative stress 3 protein that was up-regulated in ZT0 but down-regulated in ZT20 (Fig. 3).

Eighty other genes were expressed in early and late morning (ZT0–ZT4) and again in ZT20. Equivalent gene expression profiles were observed at these time periods, as exemplified by *Glyma03g37140* (outer membrane tryptophan-rich sensory protein) (TSPO)-related, *Glyma05g20400* (amino acid kinase family protein), *Glyma06g02380* (DC1 domain-containing protein), *Glyma06g45043* (O-methyltransferase 1), *Glyma06g45046*, *Glyma06g45050*, *Glyma14g32430* (highly ABA-induced PP2C gene 3), and *Glyma17g13720*, whose expression peaks reached their highest induced levels in ZT4 (Fig. 3). Alternatively, *Glyma15g40070*, which codes for a 9-*cis*-epoxycarotenoid dioxygenase 3 (NCED3) enzyme, exhibited a decreasing expression pattern between the light and dark periods (Fig. 3), although it maintained its up-regulated profile. *Glyma17g14830* (nitrate transporter 1.1), *Glyma19g34380* (indole-3-acetic-acid-inducible 14), and *Glyma02g00760* were up-regulated at midday (ZT4) but down-regulated in ZT16 (Fig. 3).

No genes in common were detected for all time periods; however *Glyma08g14670* and *Glyma20g29770* were up-regulated in five out of the six sampling times, the exception being the ZT8 period (Fig. 2). Interestingly, the expression levels of these two genes were higher in ZT0 and ZT4compared to the other periods (Fig. 3). Similar expression profiles were observed for *Glyma01g07390* (an ABI 5-binding protein), *Glyma03g24320* (fatty acid hydroxylase superfamily), and *Glyma19g11770* (a highly ABA-induced PP2C gene 2) in ZT0 − ZT4 − ZT16 − ZT20 (Fig. 3). During these same time periods, we found seven other genes that were up- or down-regulated. However, no contrasting changes were observed among these periods.

Some genes (*Glyma04g04050* [BRI1 kinase inhibitor 1], *Glyma02g05050* [eukaryotic aspartyl protease family protein], *Glyma08g15450*, *Glyma20g33740* [LRR- and NB-ARC-domain-containing disease-resistance protein], and *Glyma13g44850* [leucine-rich receptor-like protein kinase family protein]) were down-regulated in ZT0 and up-regulated in ZT12, a transition light–dark period (Fig. 3). Likewise, the transcript *Glyma04g10050* (MSCS-like 3) identified in ZT0–ZT8–ZT20 was down-regulated (-43.94 fc; ZT0) in the early morning, up-regulated later during the day (31.26 fc; ZT8), and down-regulated at the end of the dark period (-30.79 fc; ZT20) (Fig. 3), showing oscillating expression during the day. In addition, an important water-deficit related gene (*Glyma08g18801* [9-*cis*-epoxycarotenoid dioxygenase 5] [NCED5]) was dynamically up-regulated over time (ZT0 − ZT4 − ZT12 − ZT16), showing higher differential expression levels in ZT0 and ZT4 periods compared to ZT12 and ZT20 (Fig. 3). A list comparing all genes identified as differentially expressed in all time periods is presented in Additional file 2.

### Functional roles of differentially expressed soybean genes in response to water deficit

**Fig. 4:**
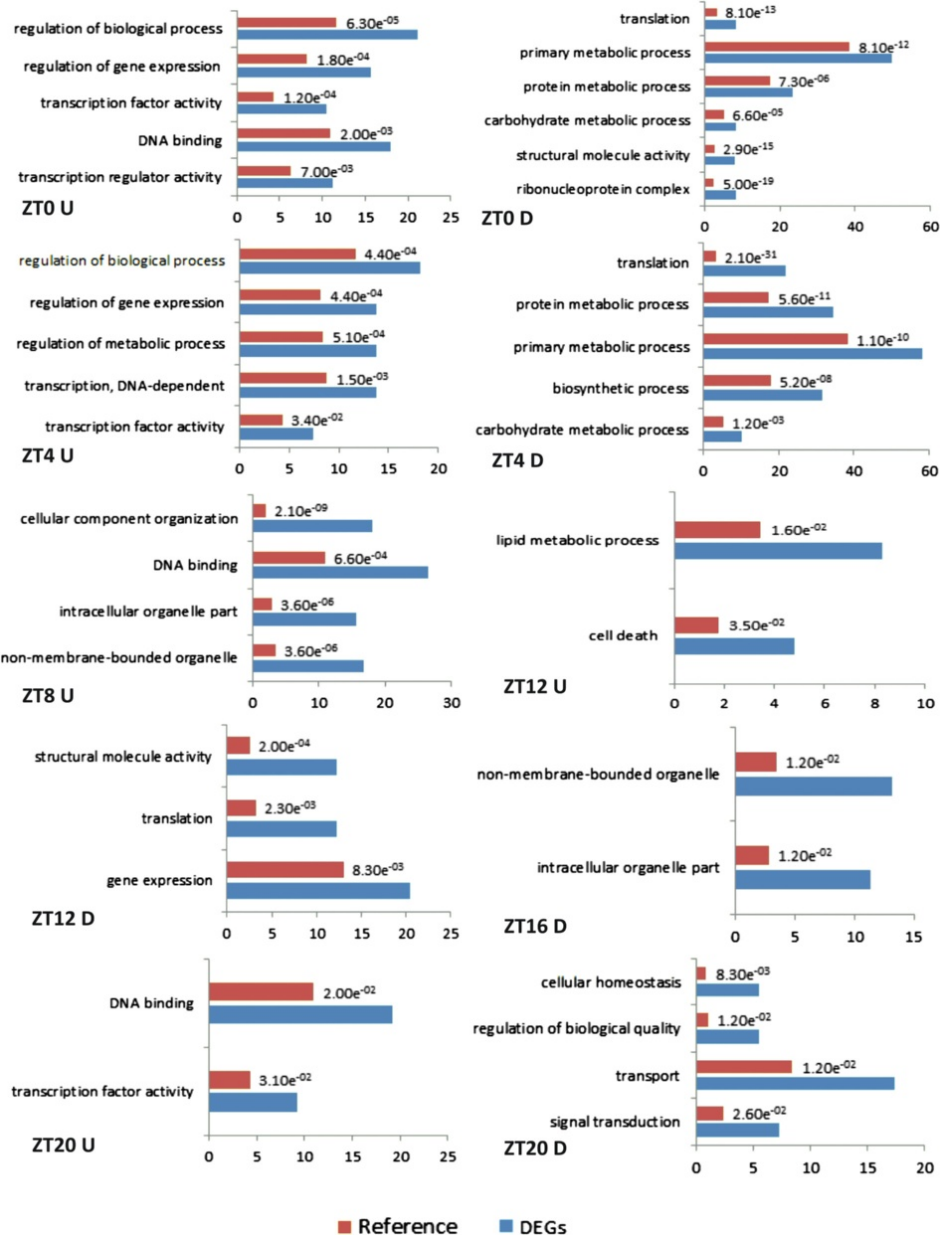
Enrichment analyses of functional roles. Genes were associated with Gene Ontology terms (Biological Process, Molecular Function and Cellular Component) and compared to the soybean genome (False Discovery Rate [FDR], *p*-value < 0.05) using AgriGO and REVIGO. Main enriched processes are presented for each time period. Red bars (Reference): genes present in soybean genome; Blue bars (DEGs): differentially expressed genes

Gene Ontology (GO) terms were associated with the DEGs to assess their putative biological roles. We performed an enrichment analysis of such terms comparing the list of DEGs identified in each time point with the annotation of the entire soybean genome (Fig. 4, Additional file 3). Initially, we found a set of 165 GO terms enriched among time periods (ZT0 − ZT20), including Biological Process, Molecular Function and Cellular Component terms. Aiming to summarize the GO terms obtained, the resulting lists were analyzed by REVIGO method [23] to remove redundant GO terms. Approximately 50 % of the processes expressed by soybean plants under water deficit were enriched in ZT0. Processes including the regulation of biological processes, transcription, and transcription factor activity were up-regulated in ZT0, whereas, among the down-regulated genes, the enriched processes were primarily represented by translation and metabolic processes (Fig. 4). Likewise, the same processes observed in both down- and up-regulated genes in ZT0, among others, were enriched in ZT4 (Fig. 4). In the ZT8 time period, there were no enriched processes for down-regulated genes, but DNA binding and processes related to cellular component organization were significantly represented among up-regulated genes (Fig. 4). Lipid metabolic processes were enriched in ZT12, while translation and structural molecular activity were down-regulated (Fig. 4). In ZT16, no enriched processes were detected for up-regulated genes, and only processes related to cellular-component terms were detected among down-regulated genes (Fig. 4). Interestingly, for genes expressed in ZT20 (pre-dawn), we observed that transcription factor activity and DNA metabolism were the only two significant processes enriched in 28.4 % of the annotated genes, the same processes enriched during light periods (ZT0 − ZT8). All enriched processes observed during the 24-h time course are described in Additional file 3.

### Diel oscillations in the expression of soybean genes under water deficit

**Fig. 5:**
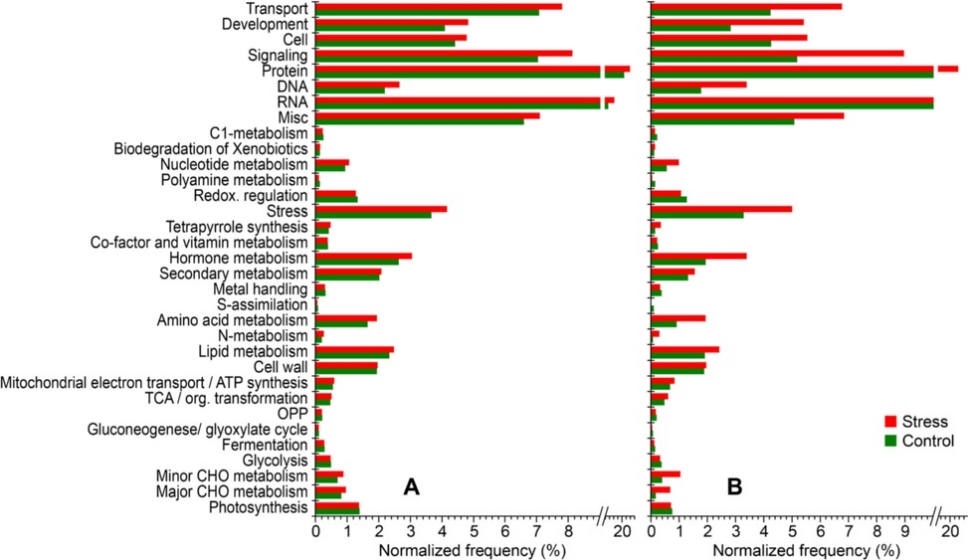
Functional classification of genes with oscillating expression in control and stressed plants. Reads per kilobase per million (RPKM) values were analyzed using the JTK Cycle algorithm to detect cycling waveforms of gene expression. The number of genes detected in each class was normalized using the total number of genes of the stress dataset. Of the total number of genes, 31.12 % (control) and 32.85 % (stress) were attributed to hypothetical pathways by associating the similarity of motifs and domains with other plant genes described in various databases (class not shown in figures). Approximately 35 % of the genes were not assigned to a pathway for both the control and stressed plants. **a** Total genes detected in each control (*n* = 19,240) and stressed plants (*n* = 21,248). **b** Genes found exclusively in the control (*n* = 4756) and stress conditions (*n* = 6764)

Expression data (reads per kilobase per million [RPKM]) from control plants and those under water deficit were collected separately to determine the putative rhythmicity of gene expression. Rhythmic waveforms were detected for 19,240 and 21,248 genes in control and water deficit plants (false discovery rate [FDR] correction, adjusted *p*-value < 0.05), respectively, which correspond to 35.52 % and 39.23 % of the whole genome (Glyma 1.1) (Additional files 4 and 5). As expected, functional classes were similar in both the control and treated plants since they shared the majority of the genes (*n* = 14,484). Most were organized into protein (synthesis, targeting, and degradation; 21.78 %) and RNA (regulation of transcription; 18.04 %) classes. Few differences were observed in classes including transport and signaling between the control (7.06 % and 7.02 %, respectively) and drought-stressed plants (7.79 % and 8.11 %, respectively) (Fig. 5a).

We also identified groups of genes that showed expression fluctuations exclusively in control (*n* = 4756) or water deficit (*n* = 6764) conditions (Fig. 5b, Additional file 6). Under normal water supply, genes expressed in a time-of-day-dependent manner played a role in several plant biological processes, particularly in the RNA (regulation, transcription, and splicing; 12.73 %) and protein (16.44 %) classes. Interestingly, genes associated with stress response (3.27 %) were detected only in the control plants (Fig. 5b, Additional file 6). Most of those genes are involved in the response to biotic stress (*n* = 110) and heat stress (*n* = 35). Similarly, expression of genes related to redox regulation metabolism (thioredoxin, ascorbate, glutathione, glutaredoxins, dismutase, and catalases; 1.25 %) was observed in the control plants (Fig. 5b).

The functionality of genes detected only in the water deficit condition was significantly increased (compared to control ones) in signaling (G-proteins, sugar, receptor-kinases, and leucine-rich repeat II; 8.94 %), RNA (regulation; 17.89 %), and protein (synthesis, post-translational modification, and degradation; 21.32 %) categories. Additionally, genes involved in hormone (synthesis/degradation of ABA, brassinosteroid, ethylene, jasmonate, salicylic acid, and hormone-mediated signaling; 3.37 %), amino acid (1.44 %), and lipid (degradation and beta oxidation; 2.40 %) metabolism also were more highly represented (Fig. 5b). After subjecting plants to water deficit, genes from the stress response class increased by 4.98 % and were particularly associated with cold (*n* = 110), PR-proteins (*n* = 84), and light (*n* = 77), among others (*n* = 92) (Fig. 5b, Additional file 6).

**Fig. 6:**
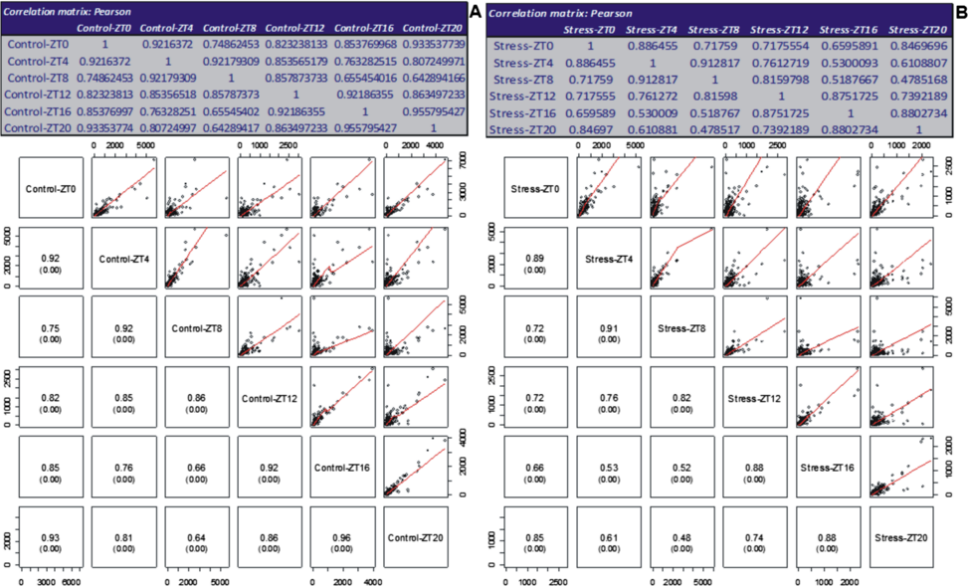
Pearson’s correlation matrix. Gene expression data (RPKM) from the (**a**) control and (**b**) stress groups were individually analyzed through pairwise comparisons to assess the similarities and dissimilarities among time periods. The matrices of scatterplots indicate the association, correlation, and *p*-value (in parentheses) of each comparison

To assess the association levels of the expression datasets (RPKM) between time periods, a matrix of similarity was calculated using the Pearson correlation coefficient (Fig. 6). Positive correlations were observed for all comparisons in the control and water deficit sets of genes at the 0.00 *p*-value. In the control plants, major differences were identified in the groups ZT0 − ZT8 (r = 0.75), ZT4 − ZT16 (r = 0.76), ZT8 − ZT16 (r = 0.66), and ZT8 − ZT20 (r = 0.64). The main dissimilarities between the water deficit treated groups occurred in the ZT0 − ZT16 (r = 0.66), ZT4 − ZT16 (r = 0.53), ZT8 − ZT16 (r = 0.52), and ZT8 − ZT20 (r = 0.48) time periods. Although expression for these sets of genes varied in plants under normal water availability, these groups decreased similarity under stress, indicating that the applied water deficit influenced the expression of those genes. Additionally, in the stressed group, the ZT0 − ZT8 (r = 0.72), ZT0 − ZT12 (r = 0.72), ZT4 − ZT12 (r = 0.76), ZT4 − ZT20 (r = 0.61), and ZT12 − ZT20 (r = 0.74) comparisons showed significant changes in the associations between different time periods (Fig. 6).

Considering the 14,484 genes shared between the control and stress conditions, 372 were specific for the stress response (2.56 %), and, of those, 198 were responsive to abiotic stress, primarily heat and drought/salt stresses (Additional file 7). This group of genes was distinct from those exclusively observed in the control or stress treatments. Although many genes specifically expressed in response to stress were detected in both control and stressed plants, it is important to emphasize that these results represent gene expression data derived from RPKM values. Since such genes appeared exclusively in control or stressed plants, the ratio of differential gene expression could not be analyzed using the software package edgeR; thus, it was not possible to infer about their up- or down-regulation under water deficit condition. According to the rhythmicity analysis of the 14,484 common genes, 12.64 % of the genes under water deficit shortened their predicted period of expression (PER) from 24 to 20 h (*n* = 1247) or from 24 to 22 h (*n* = 584) (Additional file 7). Similarly, 56.80 % of the common genes also shifted their phase (predicted phase) under water deficit. Most (*n* = 5995) advanced their expression phase under stress condition, modifying their peak from 20 to 22 h (in control) to 0 h (water deficit condition) (Additional file 7). Conversely, a set of 2233 genes delayed the LAG phase, showing their expression peak at 20 − 22 h in stress conditions, rather than at 0 h (control condition) (Additional file 7). Under water deficit, 6820 genes (47.08 %) reduced their expression amplitude (AMP), whereas 7663 (52.90 %) increased it (Additional file 7).

**Fig. 7:**
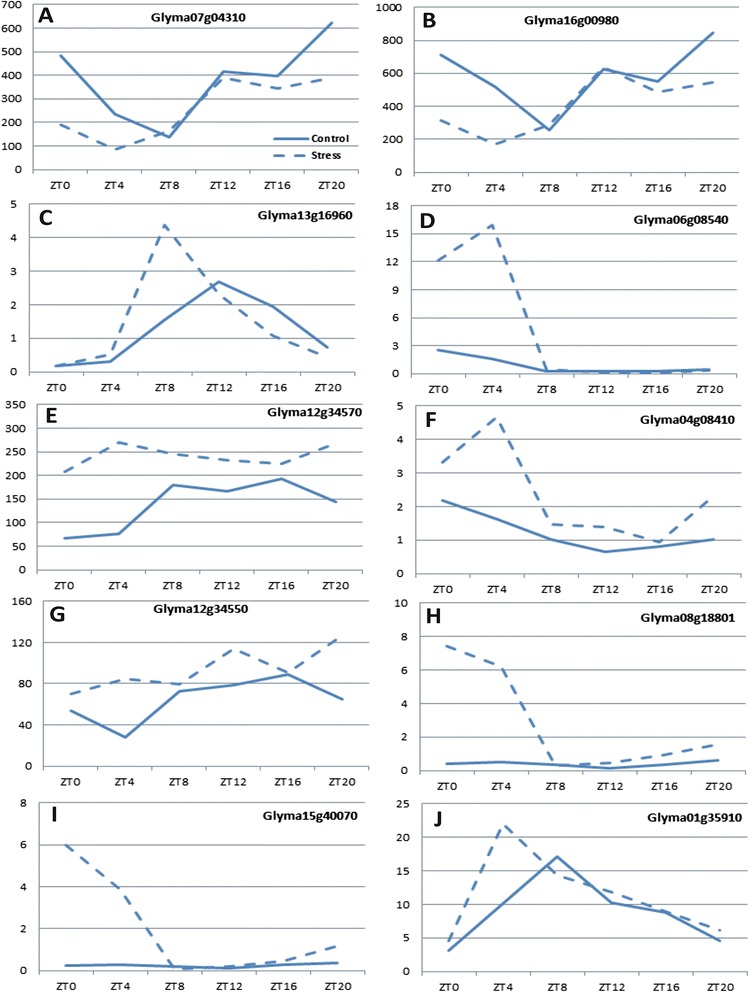
Oscillations of expression of genes observed in soybean response to water deficit stress. Waveforms were detected through rhythmicity analysis using the JTK Cycle algorithm. **a** *Glyma07g04310*, coding for germin-like protein 1; **b** *Glyma16g00980*, germin 3; **c** *Glyma13g16960*, germin-like protein 1; **d** *Glyma06g08540*, BURP domain-containing protein; **e** *Glyma12g34570*, BURP domain-containing protein; **f** *Glyma04g08410*, BURP domain-containing protein; **g** *Glyma12g34550*, BURP domain-containing protein; **h** *Glyma08g18801*, 9-*cis*-epoxycarotenoid dioxygenase 5; **i** *Glyma15g40070*, 9-*cis*-epoxycarotenoid dioxygenase 3; **j** *Glyma01g35910*, 9-*cis*-epoxycarotenoid dioxygenase 4

Some genes that showed oscillating expression were plotted on graphs to observe such patterns during the day (Fig. 7, Additional file 8). *Glyma07g04310* (coding for germin-like protein 1) and *Glyma16g00980* (germin 3) were not expressed in response to water deficit (both were down-regulated in ZT0 − ZT4; Additional file 8), but their expression was shaped by time of day in control and stressed plants (Fig. 7a and b, respectively). Similarly, *Glyma13g16960* (germin-like protein 1) also exhibited significant oscillations in both control and stressed plants, although it was induced during ZT8 (Fig. 7c). For all three genes, the PER was maintained in stressed plants, although the phase advanced and AMP was shifted, in general (Additional file 8). Oscillation patterns for genes differentially expressed during the light periods were observed for *Glyma06g08540* (BURP domain-containing protein), which exhibited notable increased expression from ZT0 (4.77 fc) to ZT4 (10.08 fc) (Fig. 7d), and for *Glyma12g34570* (BURP domain-containing protein), whose oscillation was observed specifically in control plants (Fig. 7e). *Glyma04g08410* (BURP domain-containing protein) showed differential expression at midday (2.93 fc), in ZT12 (2.32 fc) and in the dark period at ZT20 (2.34 fc) (Fig. 7f, Additional file 8). Interestingly, the expression peaks observed in these time periods were identified as differential in response to water deficit. However, overall, each control and stressed plant showed significant contrasting expression profiles (Fig. 7f). *Glyma12g34550* (BURP domain-containing protein) was up-regulated during midday (3.14 fc) and pre-dawn periods (2 fc), however, the *diel* fluctuations of expression were significant only in control plants (Fig. 7g, Additional file 8). According to the rhythmicity analysis, *Glyma08g18801* (9-*cis*-epoxycarotenoid dioxygenase 5) and *Glyma15g40070* (9-*cis*-epoxycarotenoid dioxygenase 3) showed similar expression profiles, peaking in ZT0 and ZT4 exclusively in stress condition (Fig. 7h and i, respectively) with ratios from 10 to 25 times higher (Additional file 8). Interestingly, *Glyma01g35910* (9-*cis*-epoxycarotenoid dioxygenase 4) showed a different profile under water deficit condition, with peak expression in ZT4. However, in control condition peak expression was observed in ZT8, levels that resulted in a ratio of 2.24 fc (Fig. 7j). The PER (20 h) and phase (10 h) of this oscillating expression were maintained after plants became drought-stressed, but AMP was increased from approximately 2.316 (control) to 3.639 (stress condition) (Additional file 8).

In accordance with our goals, we compared the set of genes that were differentially expressed (DEGs) in response to water deficit (Fig. 1, Additional file 1) to genes with putative cycling expression in either control or drought-stressed plants (Fig. 5, Additional files 4 and 5). Among the 4866 DEGs in response to water deficit, 3791 (3040 unique genes) were observed with oscillating expression in at least one analyzed condition. In ZT0, a large set of differentially expressed genes was composed of more down- (*n* = 1353) than up-regulated genes (*n* = 478) (Additional file 8). In ZT4, we observed the opposite expression pattern, with 674 up- and 374 down-regulated genes (Additional file 8). During the ZT8 (126 up- and 21 down-regulated) and ZT12 (248 up- and 39 down-), oscillations in gene expression occurred predominantly for up-regulated genes (Additional file 8). In the midnight period (ZT16), the pattern was similar to the pattern from early morning (ZT20), showing more down- (*n* = 79) than up-regulated genes (*n* = 39) (Additional file 8). Conversely, there was a balanced set of genes in the pre-dawn (ZT20) period, with 193 up- and 167 down-regulated genes (Additional file 8). Regarding the ZT0 − ZT8 periods, 250 genes overlapped within ZT0 − ZT4. However, approximately 96.6 % of the genes from ZT8 were uniquely expressed. Few genes exhibited overlapped expression within ZT12 − ZT20 (*n* = 8) and ZT16 − ZT20 (*n* = 10). No genes were found between ZT8 and ZT12. Of the 138 expressed genes identified in common to both the light and dark periods, 78 were expressed in the early morning (ZT0) and also detected at the pre-daw period (ZT20).

**Fig. 8:**
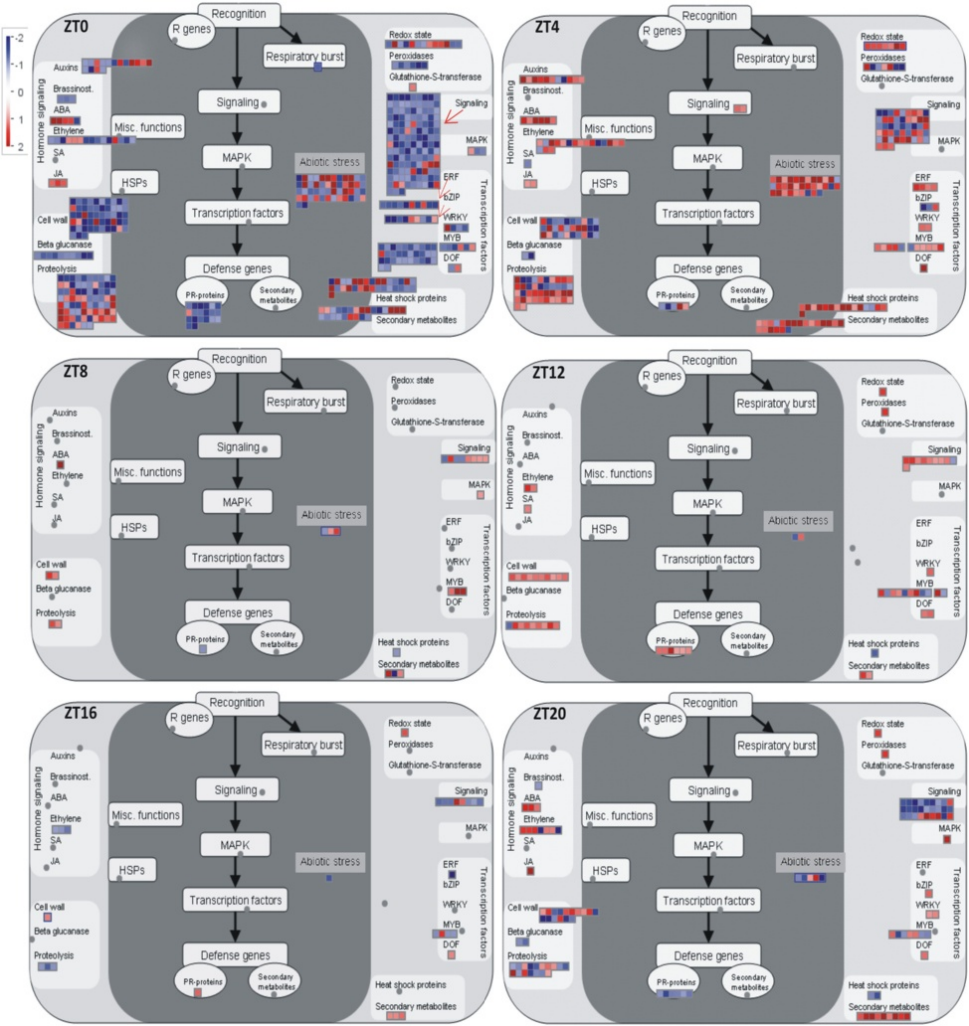
Functional roles triggered in soybean plants under water deficit stress. Genes that were differentially expressed in response to water deficit and that exhibited oscillations during the time periods analyzed were mapped to specific stress-related pathways. The color scale shows the log_2_ fold change: red = up-regulated and blue = down-regulated

All DEGs that exhibited oscillating expression (Additional file 8) were mapped to the main pathways involved in plant response to stress (Fig. 8, Additional file 9). Genes expressed in ZT0 were included in many pathways, including signaling and cell wall metabolism, that were repressed in response to water deficit during this time period. Conversely, some genes from the abiotic stress class were highly induced in a similar manner to hormone (ABA) signaling pathways and heat-shock proteins (HSPs) (Fig. 8, Additional file 9). The jasmonate pathway was also induced, as indicated by the expression of three genes related to lipoxygenase (*Glyma07g00920*), allene oxide synthase (*Glyma07g21100*), and 12-oxo-PDA-reductase (*Glyma14g39790*). The jasmonate pathway remained induced in ZT4 but was represented by two other genes encoding allene oxide synthase (*Glyma11g13070*) and 12-oxo-PDA-reductase (*Glyma11g00980*). *Glyma07g00920*, which was moderately expressed in the early morning (ZT0), also was highly up-regulated during pre-dawn (ZT20) (Fig. 8, Additional file 9). According to these results, some genes detected in a specific pathway were not expressed during the entire *diel* period, but genes encoding different proteins seem to be involved in the same stress-related pathways. The expression of genes related to PR-proteins is another example of how different genes are involved in maintaining increased or decreased metabolic activity, since most genes were distinct in each time period, and only one down-regulated gene at dawn (ZT0) (*Glyma20g33740*) was positively regulated in ZT12 (transition light–dark). All other genes related to PR-proteins in ZT12 were exclusively detected at this time period (Fig. 8, Additional file 9). In the abiotic stress class (heat, cold, drought, salt, and wounding), genes were predominantly up-regulated (expression ranged from 6.05 to 14.05 fc). Similar profiles were observed for redox-state metabolism; HSPs; ABA and other genes involved in hormone signaling; the transcription factors MYB, WRKY, DOF, and ERF; and for the class of secondary metabolites. Because the sets of DEGs were smaller in the ZT8 and ZT16 time periods, the profiles observed for the metabolic pathways were under-represented. In ZT8, few genes related to signaling and hormone signaling (ABA and jasmonate) were up-regulated. Conversely, such signaling and hormone signaling (ethylene) processes became repressed in ZT16. Although many genes were down-regulated during pre-dawn (ZT20), up-regulated ones were identified as involved in the processes of secondary metabolism, transcription factors, ABA and ethylene hormone signaling, and redox-state metabolism (Fig. 8, Additional file 9).

### Validation of gene expression

**Fig. 9:**
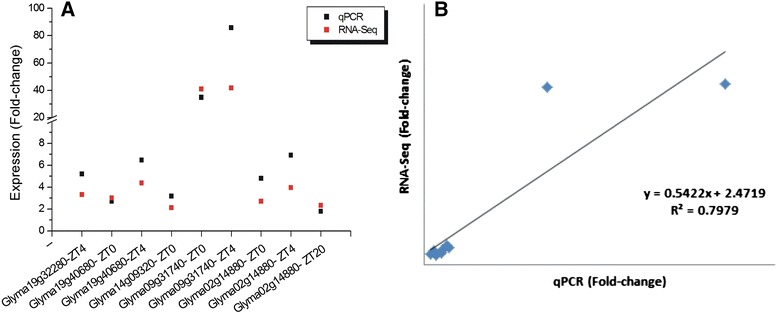
Validation of gene expression. Relative expression of the Glyma19g32280 (Remorin), Glyma19g40680 (Gols), Glyma14g09320 (DREB1), Glyma09g31740 (RAB18) and Glyma02g14880 (bZIP) was measured using the method 2^^-(ΔCt)^ in (**a**) control and drought-stressed soybean plants at specific time-periods. Glyma13g04050 (Elongation factor 1-b) and Glyma15g05570 (β-actin) were used as endogenous genes. **b** a correlation between RNA-Seq and qPCR data is shown

Expression of genes related to proteins involved in plants’ responses to water deficit stress as the Remorin (Glyma19g32280), Gols (Glyma19g40680), DREB1 (Glyma14g09320), RAB18 (Glyma09g31740) and bZIP (Glyma02g14880) were analyzed by the method 2^^-(ΔCt)^. A ratio of expression (fold-change) was calculated by dividing the expression detected in drought-stressed plants by the one observed in control (Fig. 9, Additional file 10). The gene-coding for RAB18 presented the highest fold-change in both ZT0 and ZT4 time-periods (Fig. 9, Additional file 10). In general, linear equation demonstrated a good correlation between both experiments. Genes used in this analysis represent a subset from those evaluated in Marcolino’s et al. [20] study.

## Discussion

The biological processes of plants are coordinated with physical and biochemical reactions, such as water intake, gene expression control, protein synthesis, and post-translational modification. Receptors in cell membranes communicate adverse signals from the surrounding environment to synchronize metabolic processes [7, 24, 25]. To protect themselves, plants under water deficit change their normal cellular activities, such as movement, secretion, enzyme activity, and gene expression. In *Arabidopsis,* transcriptome reconfiguration in response to drought is associated with distinct hormonal and stress response pathways induced at different times of the day [17]. Soybean genes modulating responses to water deficit stress were expressed in different time periods during the course of 24 h (Figs. 2 and 3, Additional file 2). Whereas many genes involved in translation and bioenergetic processes were repressed in most time periods, enriched processes, such as gene expression and transcription factor activity/DNA binding, were predominantly up-regulated until late day (Fig. 4) and were detected again during the pre-dawn period. Most of these transcription factors (GATA, heat shock, ERF domain, AP2, ABFs, and bZIP proteins) are directly involved in plant responses to water deficit.

Studies have demonstrated the circadian rhythm control of gene expression for different plant species [4, 5, 14–16]. In *Arabidopsis*, the number of genes under circadian regulation has been estimated to be hundreds [8, 9] to thousands regulating the control of auxin signaling [11]. Covington et al. [7] estimated that one-third of the expressed *Arabidopsis* genes are circadian clock-controlled. In maize, 10 % of the 13,000 analyzed transcripts showed circadian pattern expression [5]. Our results suggest that 35 % of the soybean genome (Glyma 1.1) showed oscillating expression in a *diel* period in plants growing under normal availability of water (Additional files 4 and 6). Seventy five percent of those genes also were detected in plants under water deficit, many of them shifted their PER or phase in such adverse condition (Additional file 5). Hsu and Harmer [24] demonstrated that differences in gene expression, even small, are associated with changes in phase and can influence expression. Additionally, dissimilarities observed in the set of expressed genes between some light/dark periods (ZT4 − ZT16, ZT8 − ZT16, and ZT8 − ZT20; Fig. 6a) reinforce the evidence of daily fluctuations in gene expression in soybean plants growing under normal conditions. Transcriptome changes observed for *Populus* submitted to drought depended on the time of day at which they were measured [16]. Similarly, in *Arabidopsis* plants, the interaction with diurnal regulation was predominant in modulating the transcriptome responses to cold stress [15]. Taken together, these data indicate that the time of day might play an important role in regulating the expression of many soybean genes in both normal and water deficit stress condition.

Gene expression changes dynamically during the course of normal development and in response to other organisms, physical damage, or adverse environmental conditions. Such behavior was observed in the expression profiles of NCED enzymes (Fig. 3), both rate-limiting components in ABA biosynthesis from the cleavage of carotenoids. In addition to the role that this phytohormone plays in normal development, ABA content is also increased in response to water deficit, preserving cell water content due to stomatal closure. In *Arabidopsis*, NCED5 acts in association with NCED3 to synthesize ABA under normal conditions and in response to stress conditions [26]. Genes related to ABA biosynthesis (including the NCEDs) have been linked to circadian regulation [7]. Interestingly, genes for NCED5 and NCED3 (Fig. 7h and i) showed higher expression levels compared to NCED genes. However, the two exhibited similar expression peaks at ZT0 − ZT4 under stress condition (but not in the control). The gene for NCED4 (*Glyma01g35910*; Fig. 7j) also was detected in the light period, but its waveforms were distinct for control (ZT8) and stress conditions (ZT4). Such changes in oscillation can be attributed to delayed amplitude under stress condition since the period and phase remained unchanged (Additional file 8). Although the diurnal fluctuation of ABA levels in tobacco plants has been implicated to occur at the end of a light period [13], as detected to the expression of NCED4 gene (Fig. 7j) in plants under normal water supply, such oscillation appeared to be limited to light under stress condition (Fig. 7h − j), the period that stomatal closure is needed to avoid water loss by evapotranspiration process.

Similarly, the gene coding for the oxidative stress 3 (OXS3) protein (Fig. 3) exhibited up-regulation in response to light (ZT0) but not dark (ZT20) (Additional file 1). This OXS3 protein is related to cadmium ion tolerance, as demonstrated in mutants of *AtOXS3* that were unable to enhance stress tolerance [27]. The authors have also associated the *AtOXS3* gene with regulation by light, suggesting a putative role for this protein in protecting the cell against photooxidation [27]. We found eight paralogs in the soybean genome that were related to the *Arabidopsis OXS3* (AT5G56550.1); three of them were identified in this study as differentially expressed in response to water deficit. In addition to *Glyma11g33040* reported above, *Glyma01g00930* and its paralog *Glyma07g15070* also were up-regulated in ZT0 (Additional file 1). Although we do not have information about the functional role of those proteins in the water deficit stress scenario, it is tempting to speculate on their importance in the soybean transcriptome since drought events can produce oxidative stress in the plants [28, 29]. Additionally, *Glyma11g33040* showed a cycling pattern when expressed in normal condition (control plants) (Additional file 1), suggesting that its expression can be influenced by time of day.

Under water deficit, it was observed that genes were predominantly down-regulated at dawn (ZT0) (Fig. 1, Additional file 1). The expression levels of 192 down-regulated genes in ZT0 did not increase at midday (ZT4) (Additional file 2). Some of these genes that exhibited down-regulated profiles in ZT4 are involved in basal metabolism, such as protein synthesis and DNA metabolism. In addition, the carbohydrate metabolic process (Fig. 4), down-regulated at dawn and midday, represented a significant change in plant bioenergetics metabolism by reducing the synthesis of organic compounds and the breakdown of carbohydrates. This suggests that during these time periods, energetically expensive processes are being partially arrested, and energy resources are being redirected to activate protective mechanisms. According to Fraire-Velázquez and Balderas-Hernández [30], the optimization of cellular energy resources during stress is essential for plant acclimation. Dhaubhadel et al. [31, 32] reported the accumulation of HSPs in *Brassica napus* seedlings under heat stress. This significant accumulation resulted from higher HSP synthesis even when the mRNA levels were lower in treated seedlings compared to controls. Such regulation mechanisms might act under post-transcriptional control, suggesting an advantageous ability of the plant machinery to save energy or drive it to maintain the translational apparatus during stress events.

In our study, the genes encoding germin and germin-like proteins are examples of genes that were repressed in early morning until midday (ZT0 − ZT4). Germin has been associated with many processes important for plant development and defense [33–35]. In the soybean genome, 36 genes annotated as germin or germin-like proteins are associated with the cupin superfamily, which includes a variety of enzymes and non-enzymatic seed-storage proteins. We detected the paralogous *Glyma07g04310* and *Glyma16g00980* down-regulated in both ZT0 and ZT4, which are associated, by suggestive evidence, with nutrient-reservoir activity. Conversely, *Glyma13g16960* (ZT8) and *Glyma01g04450* (ZT16) were up-regulated under water deficit (2.82 fc and 2.27 fc, respectively) (Additional file 1). Germin-like protein genes (Fig. 7a − c) exhibited similar oscillation profiles with respect to control plants since they all showed expression peaks in ZT12. In general, under stress, genes advanced phases and shifted amplitude. The results obtained for *Glyma13g16960* (Fig. 7c, Additional file 1) indicate that this gene is involved in the response to water deficit stress but also indicate that different germin-like protein genes expressed in soybean growth and development might oscillate levels with the time of day. The diverse expression patterns of germin-like protein genes during soybean development also were reported by Lu et al. [36]. Their study demonstrated that these genes are involved in enhancement of salt tolerance, and they exhibit expression fluctuations in darkness, suggesting a circadian clock feature [36]. In *Arabidopsis*, the ortholog (AT1G72610.1 [germin-like protein 1]) of *Glyma16g00980* as well as *Glyma07g04310*, as other germin-like proteins (GLP2 and GLP3), are associated with the extracellular matrix, hypothetically acting in developmental processes or stress responses [37]. Additionally, the circadian regulation for *Atger3*, a germin-like cell wall protein from *Arabidopsis*, seems to occur at the beginning of the night [38]. In *Sinapis alba*, a long-day plant from the same family of *Arabidopsis*, circadian oscillation was associated with a transcript that encodes a germin-like protein that exhibits a transcription peak during dark periods (ZT12 − ZT16) [39]. Likewise, circadian regulation also was suggested for the germin-like protein 9 (GLP9) in *Arabidopsis* [40].

Conversely, genes that were positively regulated in response to water deficit, such as the BURP domain-containing protein (Fig. 7d − g), were detected in various time periods (ZT0, ZT4, ZT12, and ZT20). This class of protein contains a conserved domain found in diverse plants and is putatively involved in the localization of proteins within the cell wall matrix through association with a structural domain that might target sites for intermolecular interaction [41]. Although the function of many BURP proteins is unknown, specific elements have been characterized, and the functional role of these proteins is associated with normal plant metabolic processes, such as seed development [42]. Moreover, the genes encoding the BURP domain-containing proteins also are involved in rice responses to abiotic stresses [43]. Some members of the rice BURP family are responsive to cold, ABA, and drought and salt stresses and can be induced by a single stress condition or combination of treatments. This family also exhibits temporal and spatial expression pattern differences [43]. In soybean plants, the BURP family contains 23 genes that have been classified into five subfamilies (BNM2, USP, RD22, PG1β, and BURP V). Although these genes possess no tissue specificity, they are expressed in response to stress. In particular, genes from the RD22 subfamily are the most responsive to ABA, PEG treatments, and salt stress [44]. For the GmRD22 protein (*Glyma06g085400*), the BURP domain seems to have an important role for determining its apoplast localization [45]. Furthermore, this protein-coding gene (*GmRD22* [*Glyma06g085400*]) exhibits potential responses to salt and osmotic stresses [45]. In this study, we identified four up-regulated BURP genes from the classes previously determined by Xu et al. [44]: *RD22* (*Glyma06g08540* and *Glyma04g08410*), *USP* (*Glyma12g34570*), and *Glyma12g34550* (a gene not included in the classification). Expression levels exhibited by these genes indicate the responsiveness of BURP-domain-containing proteins to water deficit, and the oscillation patterns detected in plants under normal development show the regulation of gene expression also might be influenced by time of the day.

The DEGs that showed oscillating expression in control or drought-stressed conditions (Additional file 8) played functional roles as regulatory genes in hormone signaling, cell communication, and abiotic stress-related pathways (Fig. 8, Additional file 9). Few genes involved in biotic stress responses (signaling and PR-proteins) also were down- or up-regulated during this time. Genes responsive to multiple stresses are often detected in plants under adverse conditions, since a common set of biological processes triggered by genes induced in both events converge on similar downstream responses [46, 47]. Our results showed that several hormone signaling genes, including genes related to jasmonate hormone metabolism, were observed in the early and late morning (ZT0 and ZT4), and redox reactions were up-regulated in most of the time periods (Additional file 9). Increased hormone signaling and fluctuations in the cellular redox status have been associated with plant responses to stress, and, in addition, circadian regulation also has been implicated in these processes [48]. With respect to the classification of functional roles assigned to DEGs, a similar fraction of genes down- and up-regulated in the same class can be observed. For instance, *Glyma07g04310* (Fig. 7a) and *Glyma16g00980* (Fig. 7c), both coding for the same type of protein, showed distinct expression patterns at different time periods. Evaluation of gene expression performed at a single time point during the day can only provide information about the plant’s responsiveness to drought during that specific period in which plants were sampled. In this context, soybean gene networks induced in response to stress and modulated over a *diel* period appear to be a significant feature in the acclimation process, and exploring these interactions might provide novel insight into how plants respond to water deficit.

## Conclusion

Changes in the gene expression profile of soybean leaves triggered in response to water deficit stress were dynamically modulated in the *diel* period. Such results demonstrated the importance of analyzing different time periods to characterize plant responses to stress. Analysis of rhythmicity indicates that many putative cycling genes are expressed in soybean leaves under normal development. When plants became stressed, a large number of the cycling genes found in the control plants showed a different rhythmic pattern. In addition, other genes showed fluctuations under stress conditions. Genes that were differentially expressed in more than one time period and whose expression oscillated during the course of the day provide evidence suggesting that time of day contributes to regulation of stress responses in soybean.

## Methods

### Plant growth and water deficit treatment

Soybean plants from cultivar BR16 were cultivated until the V1 developmental stage [49] in a growth chamber under specific conditions. Seeds were germinated in SuperSoil® (Scotts Miracle-Gro Company, Marysville, Ohio) at a temperature of 28/20 °C (day/night, respectively), relative humidity of 80 %, and photoperiod of 14 h day (under 500 μmol m^−2^ s^−1^ of white light)/10 h night. The experimental design was completely randomized and included two treatments (control and water deficit), six time points, and six biological replicates for each treatment/time point. Soybean field capacity was determined using the gravimetric humidity (GH) method to establish the percentage of water in the soil [50]. We previously estimated the water volume needed to reach a 100 % soil field capacity by weighting the pots daily to calculate the ratio between its fresh and dry weight. We also performed same analysis in plants after withholding irrigation to evaluate the decrease of the soil field capacity under such chamber growth conditions.

We established a 70 % soil field capacity to grow all plants during 14 days. At the 15^th^ day irrigation was suspended to initiate the water deficit treatment: control plants were maintained at 70 % and the stressed-plants were monitored periodically until soil field capacity reach 30 %, approximately (3 days after starting the stress treatment). At this condition, leaves were sampled in six time-points with consecutive 4-h intervals. Plants’ harvesting initiated at 8:00 h am (ZT0/dawn) and followed as: 12 h am/ZT4, midday; 16 h/ZT8, late day; 20 h/ZT12, transition light–dark; 24 h/ZT16, midnight; and 4 h/ZT20, pre-dawn. By convention, Zeitgeber time (ZT times) was used to indicate when light period started (ZT0) and to associate the six time-points to the respective time of day. Leaves were collected, immediately frozen in liquid nitrogen and stored at -80 °C.

### Library construction and sequencing run

Total RNA was extracted from leaves using the RNA Plant Reagent® according to the manufacturer’s instructions (Ambion, Austin, TX) and treated with DNAse (Invitrogen, Carlsbad, CA). Following analysis of RNA quality and integrity in a Bioanalyzer (Agilent, Palo Alto, CA) (only samples with a RIN ≥ 8.0 were used), equimolar quantities of purified total RNA samples from each of two biological replicates were pooled into one template for library synthesis. For each time period/treatment were synthesized three independent libraries. The Ovation RNA-Seq® (NuGEN Technologies, San Carlos, CA) method was used to enrich cDNA libraries for coding and regulatory sequences [51]. Moreover, this method was suitable for use with the Illumina platform since it permits the application of barcodes to the libraries for a multiplex sequencing strategy. Briefly, approximately 150 ng of total RNA was mixed with the selected primers to synthesize the first strand of the cDNA using a reverse transcription polymerase. The second strand was synthesized using a single primer for reverse transcription and incorporation of analog nucleotides, followed by RNA strand degradation. Double-stranded DNA was fragmented by sonication to a median size of 200 bp and purified with the Agencourt RNAClean XP system (Beckman Coulter, Brea, CA) using magnetic beads. Fragment ends were repaired to produce blunt ends and ligated to a pair of double-stranded fragments containing nucleotide analog-tagged adaptors. Strand selection eliminated sequences with the nucleotide analog (insert or insert + adaptor), thereby creating a sequence library with a single insert orientation. Sequences containing both adaptors were amplified using forward and reverse primers for 15 cycles, resulting in a specific-strand, rRNA-depleted cDNA library. Following qualitative and quantitative analysis using a Bioanalyzer, libraries were used to produce clusters for a 2 x 50 bp paired end-sequencing run. The 36 libraries were distributed into 5 lanes on a flow cell for sequencing in a Hi-Seq 2000 (Illumina). The raw data were uploaded to the GeneSifter database (Geospiza, Seattle, WA) for alignment with a reference genome.

### Mapping of reads and transcripts analysis

Base calling of the raw data was performed with parsing reads according to the respective barcodes and trimmed to remove adaptors and primer sequencing. Output sequences were aligned with the soybean genome [22] (Glyma v1.1 from the Soybase database [http://www.soybase.org]) using the BWA method [52]. For an additional alignment, a post-processing toolset (Picard; http://broadinstitute.github.io/picard/) was then used to perform local realignment, duplicate marking, and score recalibration to generate a final aligned set of genomic reads. As mapping included both unique and multiple reads, we counted these two types differentially. Unique reads were counted as a whole count, whereas multiple reads were counted proportionally for each location they were mapped to (up to five sites) when none of the adjacent unique reads appeared. Multiple reads mapped next to a set of unique reads mapped in one location were counted fully to that site. From this alignment, sequences were mapped on exons, introns, and intergenic regions, which were characterized as sequences outside of any annotated gene. The remaining unmapped reads were then aligned to a spliced reference created using all possible combinations of known exons to generate putative splice junction sites based on the annotation described above. These aligned data were then used to calculate gene expression by taking the total exon and known splice reads for each annotated gene to generate a count value per gene. For each library, a normalized expression value was then calculated for each gene using (1) the Reads per Mapped Million (RPM), which was calculated by taking the count value and dividing it by the number of millions of mapped reads and (2) the Reads Per Kilobase per Million (RPKM), which was calculated by taking the RPM value and dividing it by the kilobase length of the longest transcript for each gene.

### Differential gene expression

For each time point (ZT times), we applied a pairwise comparison between the control and water deficit treatment using all three libraries synthesized from plants of the same time period/treatment. In the pairwise analysis, we only used genes with more than 20 mapped reads to compare gene expression using the edgeR statistical test [53]. A ratio of expression (fold-change) was performed by dividing values of gene expression under water deficit and control conditions. We combined the statistical test with the multiple-hypothesis-testing correction method of Benjamini and Hochberg [54], which calculates the False Discovery Rate (FDR), to qualify statistically significant, differentially expressed genes by avoiding inflation of type-1 errors. Differential gene expression was considered significant at an adjusted *p*-value ≤ 0.01, and down- and up-regulation was established in the range of ≤ -2 to ≥ 2 fold-change (fc), respectively.

### Functional classification

Differentially expressed genes were functionally classified using gene ontology (GO) terms (http://www.geneontology.org) from Biological Process level 3. Functional classes were normalized by dividing the number of genes in each class by the total number of genes in each set (time period). The GO terms associated with the genes also were compared with the soybean genome (V1.1) using the AgriGO tool [55] to detect which GO terms were significantly enriched or depleted in a given comparison. Analysis was performed using the following parameters: Fisher’s test and multiple-hypothesis-testing correction through Hochberg FDR as statistical methods; significance level of an adjusted *p*-value < 0.05; and the Plant GO Slim database. To decrease redundancy, results provided by AgriGO were analyzed by the REVIGO (Reduce Visualize Gene Ontology) method [23] using small similarity (0.05), the Uniprot database, and SinRel as the semantic similarity measure.

### Rhythmicity analysis

The RPKM values for transcripts identified in control and drought-stressed plants were calculated separately (individual datasets for a 24-h time course with three biological replicates) and subjected to the JTK Cycles algorithm [56] to detect cycling transcripts. Period lengths (PER), phase (LAG), and amplitude (AMP) were measured using default parameters. Expression data obtained from control and stressed plants were loaded into MapMan pathways, and genes were placed into functional categories and biochemical pathways. Expression data from each time period for control and stressed plants also were compared to each other using the Pearson product–moment to generate a matrix of similarity and scatterplots.

### Validation of gene expression

Experimental procedures were performed as described by Marcolino-Gomes et al. [20]. Briefly, leaf tissue from plants under normal water irrigation regime and plants drought-stressed were used in total RNA extraction, DNAse treatment and in cDNA synthesis. qPCR assays were carried out with CFX Real-Time PCR Detection System (Bio-Rad, Hercules, USA) using three independent biological replicates (each one was composed by two plants) and two technical replicates. Soybean genes assayed were Glyma19g32280, Glyma19g40680, Glyma14g09320, Glyma09g31740 and Glyma02g14880, which codify to the proteins Remorin, GOLS (Galactinol Synthase 1), DREB1 (Dehydration Element Binding 1), RAB18 and bZIP, respectively. Primers used in cDNA synthesis are described in Table 1. Raw data was analyzed according to the method 2^^-(ΔCt)^ based on Livak and Schmittgen [57], applying the Glyma13g04050 (Elongation factor 1-b) and Glyma15g05570 (β-actin) as endogenous genes [58, 59].

**Table 1:** Primers used in qPCR assays

Gene_Name	Gene_ID	Forward primer (5′-.3′)	Reverse primer (5′-.3′)
GmRemorin-like	Glyma19g32280	TGGATTGCAGTAAGCAGCAC	AGCGTGACACCACTTATCACA
GmGOLS-like	Glyma19g40680	ACGGGGAAGGAAGAGAACAT	TGCACTCATCAATGGCTTGT
GmDREB1-like	Glyma14g09320	GATGATGATGCCTCGGAGTTG	CGGAAAAACAAGAAAAGGGATATATC
GmRAB18-like	Glyma09g31740	CAACTGGTGGCACTGGTTATGG	TGGTCATGCTGACGATGTTCCT
GmbZIP	Glyma02g14880	TAATGGGAATGGGAATTTGGG	GTTGGTGTTGGTGTTGGTGTTGTG

Gene sequences were searched in the Phytozome database and primers were designed using the PrimerQuest tool (Integrated DNA Technologies, Coralville, IA), from the 3′ untranslated region with the default settings

### Data access

The RNA-seq data discussed in this publication have been deposited in NCBI’s Gene Expression Omnibus (GEO; http://www.ncbi.nlm.nih.gov/geo/) repository and are accessible through GEO Series accession number GSE69469.

## Additional files

Additional file 1:
**Tab-delimited file showing the genes differentially expressed under water deficit conditions.** Data were analyzed for each time period using the edgeR statistical test. Genes that were up- (Up) and down-regulated (Down) are shown along with the ratio of differential expression, description, adjusted *p*-value cutoff (Benjamin and Hochberg False Discovery Rate [FDR] at *p* ≤ 0.01), and the identifying numbers from the Kegg and Gene Ontology (GO) databases. Data from the rhythmicity analysis are also presented. (Adj. P) adjusted *p*-value ≤ 0.05; (PER) predicted period (h); (LAG) predicted phase (h); and (AMP) reports amplitude. Numbers in the control and stress datasets represent the expression levels (in RPKM). Additional file 2:
**Tab-delimited file showing the genes exclusively expressed or overlapping in different time periods.** The gene list was organized from Edwards’s diagram (Fig. 2). Genes that were up- (Up) and down-regulated (Down), the ratio of differential expression, and the description are presented. Additional file 3:
**Tab-delimited file showing all enriched processes observed in each time period.** Gene ontology (GO) terms were compared to the soybean genome using a statistical cutoff (False Discovery Rate [FDR], *p* < 0.05). Enrichment analyses were performed using the AgriGO database and REVIGO method. All processes (Biological Processes, Molecular Function, Cellular Component) considered enriched in each time period are shown. Additional file 4:
**Tab-delimited file showing the rhythmicity analysis of the transcripts expressed in control plants.** Expression data were analyzed using the JTK Cycle algorithm to detect cycling transcripts. (Adj. P) adjusted *p*-value ≤ 0.05; (PER) predicted period (h); (LAG) predicted phase (h); (AMP) amplitude. Numbers in the control and stress datasets represent the expression levels (in RPKM). Additional file 5:
**Tab-delimited file showing the rhythmicity analysis of transcripts expressed in stressed plants.** Expression data were analyzed using the JTK Cycle algorithm to detect cycling transcripts. (Adj. P) adjusted *p*-value ≤ 0.05; (PER) predicted period (h); (LAG) predicted phase (h); (AMP) amplitude. Numbers in the control and stress datasets represent the expression levels (in RPKM). Additional file 6:
**Tab-delimited file showing the genes from the rhythmicity analysis that were detected in both control and stressed -plants or exclusively in each condition.**
Additional file 7:
**Tab-delimited file showing the rhythmicity analysis of transcripts common to control and drought-stressed plants.** Expression data were analyzed using the JTK Cycle algorithm to detect cycling transcripts. (Adj. P) adjusted *p*-value ≤ 0.05; (PER) predicted period (h); (LAG) predicted phase (h); (AMP) amplitude. Numbers in the control and stress datasets represent the expression levels (in RPKM) and the means of three biological replicates. The PER (blue area, left axis), LAG (red area, left axis), and AMP (green line, right axis) data were plotted on graphs, and genes were organized at the same positions on the x-axis. Additional file 8:
**Tab-delimited file showing all differentially expressed genes (DEGs) that showed significant oscillating expression in control or stressed plants.** Genes that were up- (Up) and down-regulated (Down) are shown along with the ratio of differential expression, description, False Discovery Rate (FDR) cutoff (Benjamin and Hochberg at *p* ≤ 0.01), and the identifying numbers from the Kegg and Gene Ontology (GO) databases. (Adj. P) adjusted *p*-value ≤ 0.05; (PER) predicted period (h); (LAG) predicted phase (h); and (AMP) reports amplitude. Numbers in the control and stress datasets represent the expression levels (in RPKM). Additional file 9:
**Tab-delimited file showing the pathway mapping.** The MapMan database was searched for stress-responsive genes that showed cycling expression oscillation to identify the biochemical pathways that either were or were not activated in response to water deficit stress in soybean plants. Bincode (numeric index of the functional categories), BinName (functional categories), Gene ID, Description and log_2_ fold-change (negative values represent down-regulated genes) are shown for genes presented in Additional file 8. Additional file 10:
**Tab-delimited file showing the data of qPCR analysis. Raw data was analyzed by the method 2^** ^**-(ΔCt)**^ **.** The normalized data of each biological replicate is presented for control and drought-stressed plants. For RNA-Seq data, biological replicates were analyzed by edgeR statistical test and expression data is represented by a mean for control and drought-stressed plants. A ratio of expression (fold-change) was calculated by dividing of gene expression under water deficit and control conditions for each experiment.

## Abbreviations

PR: Pathogenesis-Related
ABA: Abscisic Acid
DREB: Dehydration Responsive Element Binding
GO: Gene Ontology
RPKM: Reads Per Kilobase per Million
RPM: Reads Per Million
FDR: False Discovery Rate
BNM2: Microspore-derived embryo from *Brassica napus*
USP: Unknown Storage Protein
RD22: Responsive to Droughtness
PG1β: β-subunit of polygalacturonidase
BURPV: BURP domain protein from class V, named according to the proteins it was first identified: BNM2, USP, RD22, and PG1β
GLP: Germin-Like Protein
NCED: Nine-Cis-Epoxycarotenoid Dioxygenase
PEG: Polyethylene Glicol
BWA: Burrows-Wheeler Alignment
ZT: Zeitgeber Time
AgriGO: GO Analysis toolkit for the agricultural community
REVIGO: Reduce Visualize Gene Ontology
PER: Period
LAG: Phase
AMP: Amplitude
